# Sleep Across the Pandemic in Norwegian University and College Students: A National Repeated Cross‐Sectional Analysis (2010–2023)

**DOI:** 10.1111/jsr.70312

**Published:** 2026-02-19

**Authors:** Børge Sivertsen, Allison G. Harvey, Øystein Vedaa, Ståle Pallesen, Mari Hysing

**Affiliations:** ^1^ Department of Health Promotion Norwegian Institute of Public Health Bergen Norway; ^2^ Department of Research & Innovation Helse Fonna HF Haugesund Norway; ^3^ Department of Psychology University of California Berkeley California USA; ^4^ Department of Psychosocial Science, Faculty of Psychology University of Bergen Bergen Norway

**Keywords:** higher education, insomnia disorder, population trends, sleep duration, social jetlag, student well‐being

## Abstract

Sleep problems are increasingly common among students in higher education, but long‐term trajectories before, during and after the COVID‐19 pandemic remain poorly described. We analysed population‐level surveillance data from the Students' Health and Wellbeing Study conducted in Norway in 2010, 2014, 2018, 2021, 2022 and 2023, including nearly 200,000 participants. Difficulties initiating or maintaining sleep were assessed in all waves using a consistent survey item. In the 2018, 2021 and 2022 surveys, participants also reported bedtime, risetime, sleep onset latency, wake after sleep onset and total sleep duration. DSM‐5‐based insomnia disorder symptoms were identified using criteria based on symptom frequency, duration and daytime impairment. The prevalence of difficulties initiating or maintaining sleep increased steadily from 2010 to 2023, rising from 23.8% to 38.3% among women and from 20.3% to 32.2% among men. Symptom prevalence was higher in every survey wave compared with 2010, with the steepest increase occurring during the pandemic. Insomnia disorder symptoms increased markedly from 2018 to 2021 and declined only modestly in 2022. Sleep onset latency and wake after sleep onset increased during the pandemic, resulting in reduced sleep efficiency, whereas mean sleep duration remained stable at approximately seven and a half hours. Bedtime and risetime were delayed during the pandemic, with partial reversion in 2022. In conclusion, worsening sleep among students appears to represent a sustained trend rather than a transient pandemic‐related disruption. These findings provide population‐level surveillance of long‐term sleep trends and underscore the need for systematic support for student sleep health within higher education.

## 1 Introduction

Sleep problems are increasingly common among university students and pose a significant concern for public health, academic functioning and overall well‐being (Gardani et al. 2022). University students are particularly vulnerable to sleep problems due to multiple interacting factors, including academic stress, irregular schedules, late‐night technology use, extracurricular work and social obligations (Gardani et al. 2022; Bjornnes et al. 2021; Barone 2017). Sleep problems in this group are further exacerbated by biological predispositions toward eveningness during young adulthood (Kjorstad et al. 2022), which may result in a misalignment between circadian rhythms and institutional demands (Rezaei and Grandner 2021).

Findings from the Norwegian Students' Health and Wellbeing Study (SHoT) (Sivertsen, Rakil, et al. 2019), a national repeated cross‐sectional survey of college and university students, have consistently documented high rates of sleep problems, with nearly one in three students reporting insomnia symptoms (Sivertsen, Vedaa, et al. 2019). Even before the COVID‐19 pandemic, average sleep duration among students was below recommended levels (Sivertsen, Vedaa, et al. 2019). During the pandemic, measures such as lockdowns, social distancing and campus closures were associated with disruptions in both sleep patterns and mental health among students (Yuksel et al. 2021; Sivertsen et al. 2025). Longitudinal studies comparing prepandemic with pandemic data suggest increased rates of problems and sleep pattern disruptions, albeit with some increase in sleep duration (Valenzuela et al. 2023). Although sleep patterns partially reverted toward prepandemic levels following the lifting of restrictions in 2022, sleep problems remained prevalent (Rezaei and Grandner 2021; Bottary et al. 2023). For example, data from SHoT2022 indicated that one third of students met criteria for insomnia disorder, with higher rates observed among women (Sivertsen and Johansen 2022).

The persistence of sleep problems in the student population is concerning, given established links with academic difficulties, impaired daily functioning and long‐term mental and physical health risks (Becker et al. 2018; Seoane et al. 2020; McMahon et al. 2019). Despite growing empirical attention, sleep problems among students remain under‐recognised and insufficiently addressed. Recent evidence emphasises the importance of treating student sleep health as a distinct public health priority, underscoring the need for tailored interventions and institutional strategies (Bjornnes et al. 2021). From a public health perspective, systematic surveillance of student sleep health is essential for identifying long‐term trends, monitoring the population‐level impact of major societal disruptions, such as the COVID‐19 pandemic, and informing policy and institutional responses. Interestingly, a meta‐analysis of psychological treatments to improve sleep among university students showed that psychological interventions outperformed control groups (Tadros et al. 2025).

The present study aims to examine long‐term population‐level trends in sleep problems among Norwegian higher education students from 2010 to 2023, using repeated cross‐sectional data from the SHoT study. Consistent with a surveillance framework, we investigated: (1) Changes in key sleep parameters, including sleep duration, sleep onset latency (SOL), wake after sleep onset (WASO), bedtime, risetime and sleep efficiency, across the 2018, 2021 and 2022 waves; (2) Trends in the prevalence of insomnia symptoms (DIMS) and DSM‐5‐based insomnia disorder symptoms across six survey waves and (3) Whether pandemic‐related changes in sleep patterns represent a temporary deviation or a sustained shift in student sleep health.

## 2 Methods

### 2.1 Study Design, Participants and Setting

The SHoT study (*Students' Health and Wellbeing Study*) is a national survey of students in higher education in Norway, initiated by the three largest student welfare organisations. So far, four main surveys of the student population (aged 18–35) have been conducted (2010, 2014, 2018 and 2022). Additionally, two smaller surveys were conducted in 2021 (during the COVID‐19 lockdown) and in 2023, with the latter focusing on mental health symptoms and disorders. All waves collected data electronically through a web‐based platform and were designed as independent, cross‐sectional surveys. Although the SHoT2023 survey recontacted a subset of participants from SHoT2022, this follow‐up was not intended to function as a longitudinal cohort and was accordingly analysed within a repeated cross‐sectional surveillance framework.

Details of the SHoT study have been published elsewhere (Sivertsen, Rakil, et al. 2019). In short, the SHoT2010 study was conducted between 11 October and 8 November 2010. The target group comprised a random sample of 26,779 Norwegian full‐time students, of whom 6053 students completed the survey, yielding a response rate of 22.6%. The SHoT2014 study was conducted between 24 February and 27 March 2014. An invitation email containing a link to an anonymous online questionnaire was sent to 47,514 randomly selected students. For the SHoT2014 survey, the sampling was stratified by study institutions, faculties and departments. The overall response rate was 28.5% and included 13,525 students. *The SHoT2018* was conducted between 6 February and 5 April 2018, inviting all fulltime Norwegian students pursuing higher education (both in Norway and abroad). For the SHoT2018 study, 162,512 students fulfilled the inclusion criteria, of whom 50,054 students completed the online questionnaires, yielding a response rate of 30.8%. *The SHoT2021* was conducted between 1 March and 6 April 2021. This was a shorter health survey focusing specifically on health outcomes during the COVID19‐lockdown. In all, 181,828 students were invited to participate, of which 62,498 students completed the survey, yielding a response rate of 34.4%. *The SHoT2022* survey was conducted between 6 March and 19 April 2022, and all full‐time students in higher education in Norway (*N* = 169,572) were invited. A total of 59,544 students completed the survey, yielding a response rate of 35.1%. As part of the consent process, participants were also invited to be contacted for a follow‐up study on mental health in 2023. Of the SHoT2022 respondents, 26,311 consented to be recontacted. To achieve a more balanced sex distribution in the follow‐up sample (due to higher female participation in the main study), a proportionally larger share of male students was reinvited. The final sample included 16,418 students who were still registered as full‐time students in January 2023. Of these, 10,460 participated in the follow‐up survey conducted between 24 January and 6 February 2023. As part of this follow‐up, all participants completed the Hopkins Symptom Checklist‐25 (HSCL‐25). The SHoT2023 data collection was originally conducted for a different primary purpose, namely to assess the prevalence of mental disorders in the student population using diagnostic interviews (Hysing et al. 2025). The HSCL‐25 was included to evaluate its ability to estimate prevalence rates of anxiety and depressive disorders relative to diagnostic interviews (Sivertsen et al. 2024). Because this instrument includes one item assessing difficulties initiating or maintaining sleep, the 2023 data provided an opportunity to extend the surveillance of sleep problems into this year. For the present study, we used data from the subgroup of participants who completed the HSCL‐25 prior to any diagnostic or additional follow‐up assessments, ensuring consistency with standard population‐based survey methodology.

All parts of the project, including the planning of research questions, selection of study questionnaires, piloting, collection of data, as well as utilisation of data and findings, were conducted in close collaboration with the student welfare organisations in Norway. The study was framed as a general survey of students' health and wellbeing and promoted through largely consistent recruitment channels across all waves, primarily direct email and SMS invitations distributed via institutional student registries and supported by student welfare organisations.

While the pandemic has had a major impact worldwide, countries differed in their response and the nature and extent of restrictions imposed, as well as mortality rates (Villani et al. 2020; GBD 2021 Causes of Death Collaborators 2024). In Norway, there was a relatively low infection rate compared to other European countries. While there were not any complete lockdowns or nationwide curfews, containment measures to restrict social contact, including the closure of or limited access to campuses and restrictions on many other services (like gyms and restaurants/pubs), were common in some regions and in most large cities. During the SHoT2021 survey period, multiple national and regional restrictions were in place, resulting in predominantly online teaching and closed campuses. By the time of the 2022 data collection, most national and regional restrictions in Norway had been lifted just prior to survey initiation. However, some restrictions remained, and students were typically offered a hybrid model of on‐campus and online teaching (Ministry of Education and Research 2022).

### 2.2 Procedure and Measures

From 2018 onwards, participants' age and sex were extracted based on their 11‐digit Norwegian national identity number, whereas in the 2010 and 2014 surveys, these data were based on self‐report.

#### 2.2.1 DIMS (2010–2023)

Sleep problems were assessed using one item from the Hopkins Symptom Checklist (Derogatis et al. 1974), included in both the full HSCL‐25 (administered in 2010, 2014, 2018, 2022 and 2023) and the abbreviated HSCL‐5 (used in the 2021 wave). This item asks participants to rate how often they have experienced ‘trouble falling asleep or staying asleep’ during the past 2 weeks. Response options range from 1 (‘not at all’) to 4 (‘extremely’). For this study, we defined DIMS as responding either ‘quite a bit’ or ‘extremely’. While the full and abbreviated HSCL versions differ in length, the sleep item itself remains identical, allowing for comparisons over time. Throughout the manuscript, DIMS refers specifically to this HSCL‐based measure.

#### 2.2.2 Detailed Sleep Measures in 2018–2022

Detailed sleep measures were included in the SHoT surveys conducted in 2018, 2021 and 2022. Participants reported their usual bedtime and risetime in hours and minutes, separately for weekdays and weekends. Time in bed (TIB) was calculated as the interval between bedtime and risetime. SOL and WASO were also reported for both weekdays and weekends. Sleep duration was defined as TIB minus SOL and WASO. In the present study, sleep duration was analysed as a continuous variable. Sleep efficiency was calculated as (sleep duration ÷ TIB) × 100 and expressed as a percentage. Social jetlag was calculated as the absolute difference between the mid‐point of sleep on free days (MSF) and on weekdays (MSW), where each mid‐point was defined as bedtime + SOL + half of sleep duration (Wittmann et al. 2006). In addition, participants were asked to indicate how many nights per week they experienced difficulties initiating sleep (DIS), difficulties maintaining sleep (DMS) and early morning awakenings (EMA), as well as how many days per week they experienced daytime sleepiness and/or tiredness. For those reporting sleep problems, duration was also assessed. DSM‐5 based insomnia disorder symptoms were operationalised using a symptom definition adapted for epidemiological surveys, requiring: (Gardani et al. 2022) the presence of DIS, DMS or EMA occurring ≥ 3 nights per week, (Bjornnes et al. 2021) daytime sleepiness or tiredness occurring ≥ 3 days per week, and (Barone 2017) symptom duration at least 3 months. This operationalisation captures key DSM‐5 symptom domains but does not constitute clinical diagnostics. Table 1 presents the full set of sleep items included in these survey waves.

**TABLE 1:** Descriptive characteristics of participants across all SHoT survey waves (2010–2023).

Characteristics	2010		2014		2018		2021		2022		2023		Statistics
	*n*	%	*n*	%	*n*	%	*n*	%	*n*	%	*n*	%	
Participants (response rate)	6053	22.6%	13,525	28.5%	50,054	30.8%	62,498	34.4%	59,544	35.1%	10,460	63.7%^a^	
Gender													*χ* ^2^ = 195.0 df = 5, *p* < 0.001
Men	2071	34.2%	4581	33.5%	15,398	30.9%	20,307	34.4%	19,967	33.5%	1492	29.4%	
Women	3982	65.8%	9082	66.5%	34,435	69.1%	38,718	65.6%	39,575	66.5%	3582	70.6%	
Age‐group (years)													*χ* ^2^ = 714.9, df = 20, *p* < 0.001
18–20	1237	20.4%	1767	12.9%	8831	17.9%	9555	16.1%	8494	15.9%	875	17.2%	
21–22	1711	28.3%	3678	26.9%	15,471	31.4%	18,060	30.5%	16,214	30.4%	1537	30.3%	
23–25	1921	31.7%	4887	35.8%	15,901	32.2%	20,444	34.5%	17,504	32.8%	1609	31.7%	
26–28	753	12.4%	2006	14.7%	5710	11.6%	6917	11.7%	6431	12.1%	585	11.5%	
29–35	431	7.1%	1325	9.7%	3426	6.9%	4297	7.2%	4718	8.8%	468	9.2%	

^a^ Conditional response rate based on the subsample of SHoT2022 participants who consented to recontact and were eligible for the 2023 follow‐up.

### 2.3 Statistical Analyses

All statistical analyses were performed using IBM SPSS Statistics version 30 (SPSS Inc., Chicago, IL). Estimated Marginal Means (EMMs) were calculated using the UNIANOVA procedure to assess differences in continuous sleep variables across the 2018, 2021 and 2022 surveys. These analyses were adjusted for age and stratified by sex. To evaluate changes in the prevalence of DIMS, binary logistic regression analyses were performed using the 2010 wave as the reference year. Adjusted odds ratios (ORs) and 95% confidence intervals (CIs) were estimated for each survey wave, separately for men and women, adjusting for age. Additional logistic models compared odds across the 2018, 2021, 2022 and 2023 waves to assess changes before, during and after the COVID‐19 pandemic. For Table 2, nominal *p* values were adjusted using the Benjamini‐Hochberg false discovery rate procedure, and results are presented as *q* values. All analyses were conducted at the survey‐wave level to estimate population‐level trends rather than within‐person change, consistent with the repeated cross‐sectional design of the study. Missing data were minimal across all waves and handled using listwise deletion.

**TABLE 2:** Odds ratios (OR) with 95% confidence intervals (CI) for difficulties initiating or maintaining sleep (DIMS) among university students by sex, comparing survey waves from 2010 to 2023.

Wave comparison	Female		Male	
	OR (95% CI)	*q* value	OR (95% CI)	*q* value
2010 vs. 2014	1.20 (1.10–1.31)	< 0.001	1.23 (1.08–1.40)	< 0.001
2010 vs. 2018	1.53 (1.42–1.66)	< 0.001	1.40 (1.25–1.56)	< 0.001
2010 vs. 2021	1.70 (1.58–1.84)	< 0.001	1.81 (1.62–2.02)	< 0.001
2010 vs. 2022	1.56 (1.45–1.69)	< 0.001	1.55 (1.39–1.74)	< 0.001
2010 vs. 2023	1.99 (1.82–2.17)	< 0.001	1.87 (1.63–2.13)	< 0.001
2014 vs. 2018	1.28 (1.21–1.35)	< 0.001	1.14 (1.05–1.23)	< 0.001
2014 vs. 2021	1.42 (1.35–1.49)	< 0.001	1.47 (1.37–1.59)	< 0.001
2014 vs. 2022	1.30 (1.24–1.37)	< 0.001	1.26 (1.17–1.36)	< 0.001
2014 vs. 2023	1.66 (1.55–1.77)	< 0.001	1.52 (1.38–1.68)	< 0.001
2018 vs. 2021	1.10 (1.08–1.14)	< 0.001	1.29 (1.23–1.36)	< 0.001
2018 vs. 2022	1.02 (0.98–1.05)	0.12	1.13 (1.06–1.21)	< 0.001
2018 vs. 2023	1.30 (1.23–1.37)	< 0.001	1.34 (1.23–1.45)	< 0.001
2021 vs. 2022	0.92 (0.89–0.95)	< 0.001	0.87 (0.83–0.92)	< 0.001
2021 vs. 2023	1.17 (1.11–1.23)	< 0.001	1.04 (0.98–1.11)	0.18
2022 vs. 2023	1.27 (1.21–1.34)	< 0.001	1.20 (1.11–1.31)	< 0.001

*Note: q* values are adjusted for multiple testing using the Benjamini‐Hochberg false discovery rate (FDR) procedure across all comparisons in Table 2.

### 2.4 Ethics

All procedures involving human subjects/patients were approved by the Regional Committee for Medical and Health Research Ethics in Western Norway (SHoT2018: no. 2017/1176, SHoT2021: no. 176205 and SHoT2022/2023: no. 326437, respectively). Approvals for conducting the SHoT2010 and SHoT2014 studies were granted by the Data Protection Officer for research at the Norwegian Centre for Research Data. Electronic informed consent was obtained after presenting a complete written description of the study to the participants.

## 3 Results

### 3.1 Sample Characteristics

Data from the SHoT surveys conducted between 2010 and 2023 indicated gradual demographic shifts among student participants. As presented in Table 1, the proportion of female respondents increased modestly across the study period. Age distribution was also altered: in 2010, students aged 18–20 made up 20.4% of the sample, but this share declined to 15.9% by 2022, suggesting an overall aging trend in the student population. The 2023 survey attested to this pattern, likely influenced by the re‐surveying of students from the 2022 cohort, who were, on average, 1 year older.

### 3.2 Trend of Sleep Characteristics

Table 3 presents descriptive statistics for key sleep parameters among female and male students across the 2018, 2021 and 2022 SHoT waves. Clear sex differences and temporal patterns were observed across all parameters. Across all years, males consistently reported later bedtimes and risetimes than females. In 2018, the average bedtime for females was 23:05 (95% CI: 23:04–23:05), compared to 23:37 (23:36–23:38) for males, a difference of 32 min. Both sexes reported delayed bedtimes in 2021, likely reflecting pandemic‐related changes in routines, with females shifting to 23:11 and males to 23:44. By 2022, bedtimes had largely returned to prepandemic levels. A similar pattern was observed for risetimes, with peak delays in 2021 (females: 08:02; males: 08:22) and partial reversion in 2022.

**TABLE 3:** Weekday sleep characteristics by sex and survey year.

Sleep characteristic	Female			Male		
	2018	2021	2022	2018	2021	2022
Bedtime	23:05 (23:04–23:05)	23:11 (23:10–23:12)	23:03 (23:02–23:04)	23:37 (23:36–23:38)	23:44 (23:43–23:45)	23:32 (23:31–23:33)
Risetime	07:37 (07:36–07:38)	08:02 (08:01–08:03)	07:42 (07:41–07:43)	07:59 (07:57–08:00)	08:22 (08:20–08:23)	08:00 (07:59–08:01)
Social jetlag	1:55 (1:54–1:55)	Not available	1:43 (1:42–1:43)	1:54 (1:53–1:55)	Not available	1:40 (1:39–1:41)
Sleep onset latency	0:50 (0:49–0:50)	0:59 (0:58–0:59)	0:51 (0:51–0:52)	0:43 (0:42–0:44)	0:54 (0:53–0:54)	0:46 (0:46–0:47)
Wake after sleep onset	0:16 (0:16–0:16)	0:18 (0:18–0:19)	0:20 (0:19–0:20)	0:13 (0:12–0:14)	0:15 (0:15–0:16)	0:17 (0:16–0:17)
Time in bed	08:32 (08:31–08:33)	08:50 (08:50–08:51)	08:38 (08:38–08:39)	08:21 (08:20–08:22)	08:37 (08:36–08:38)	08:27 (08:26–08:28)
Sleep duration	07:25 (07:24–07:26)	07:32 (07:32–07:33)	07:27 (07:26–07:27)	07:24 (07:23–07:25)	07:27 (07:26–07:28)	07:24 (07:22–07:25)
Sleep efficiency	86.9% (86.8–87.1)	85.4% (85.3–85.6)	86.2% (86.1–86.3)	88.6% (88.4–88.8)	86.7% (86.5–86.8)	87.5% (87.3–87.7)

Social jetlag, measured in 2018 and 2022, averaged close to 2 h and was higher among females. In 2018, females reported 1:55 compared to 1:43 among males. In 2022, averages were 1:54 for females and 1:40 for males.

SOL increased from 2018 to 2021 in both sexes, for females rising from 50 to 59 min, and for males from 43 to 54 min. By 2022, a modest decline was observed (to 51 min for females; 46 min for males), but values remained higher than in 2018. WASO followed a similar upward trend. Among females, WASO increased from 16 min (2018) to 20 min (2022), while for males this parameter increased from 13 to 17 min over the same period.

TIB increased from 2018 to 2021, coinciding with lockdown periods, before slightly declining in 2022. For females, average TIB was 08:32 in 2018, 08:50 in 2021 and 08:38 in 2022. For males, TIB increased from 08:21 in 2018 to 08:37 in 2021 and declined to 08:27 in 2022. Despite this fluctuation in TIB, sleep duration remained relatively stable. Females reported 07:25 (2018), 07:32 (2021) and 07:27 (2022), respectively; males reported 07:24 across all 3 years, with only minor variation.

Sleep efficiency declined from 2018 to 2021, reflecting longer SOL and WASO, and only partially recovered in 2022. Among females, efficiency dropped from 86.9% (2018) to 85.4% (2021), then rose to 86.2% (2022). Among males, sleep efficiency decreased from 88.6% to 86.7% between 2018 and 2021, with a rebound to 87.5% in 2022. Across all time points, males reported consistently higher sleep efficiency than females.

### 3.3 Prevalence of Insomnia Disorder

The prevalence of DSM‐5‐based insomnia disorder symptoms differed markedly across survey waves and was consistently higher among women than men. In 2018, insomnia disorder symptoms were reported by 34.2% (95% CI 33.7–34.7) of female students, and 22.2% (95% CI 21.5–22.8) of male students. During the pandemic in 2021, prevalence increased substantially in both sexes, reaching 46.9% (95% CI 46.4–47.4) among women (18,143 of 38,718) and 35.0% (95% CI 34.3–35.6) among men (7098 of 20,307). By 2022, prevalence declined but remained elevated relative to prepandemic levels. Among women, 37.3% (95% CI 36.8–37.8) met criteria for insomnia disorder symptoms (14,771 of 39,574), compared with 25.8% (95% CI 25.2–26.4) among men (5156 of 19,967). Across all three waves, women consistently reported higher prevalence than men, and the largest increase occurred between 2018 and 2021, followed by only partial normalisation in 2022.

### 3.4 Prevalence of DIMS

**FIGURE 1:**
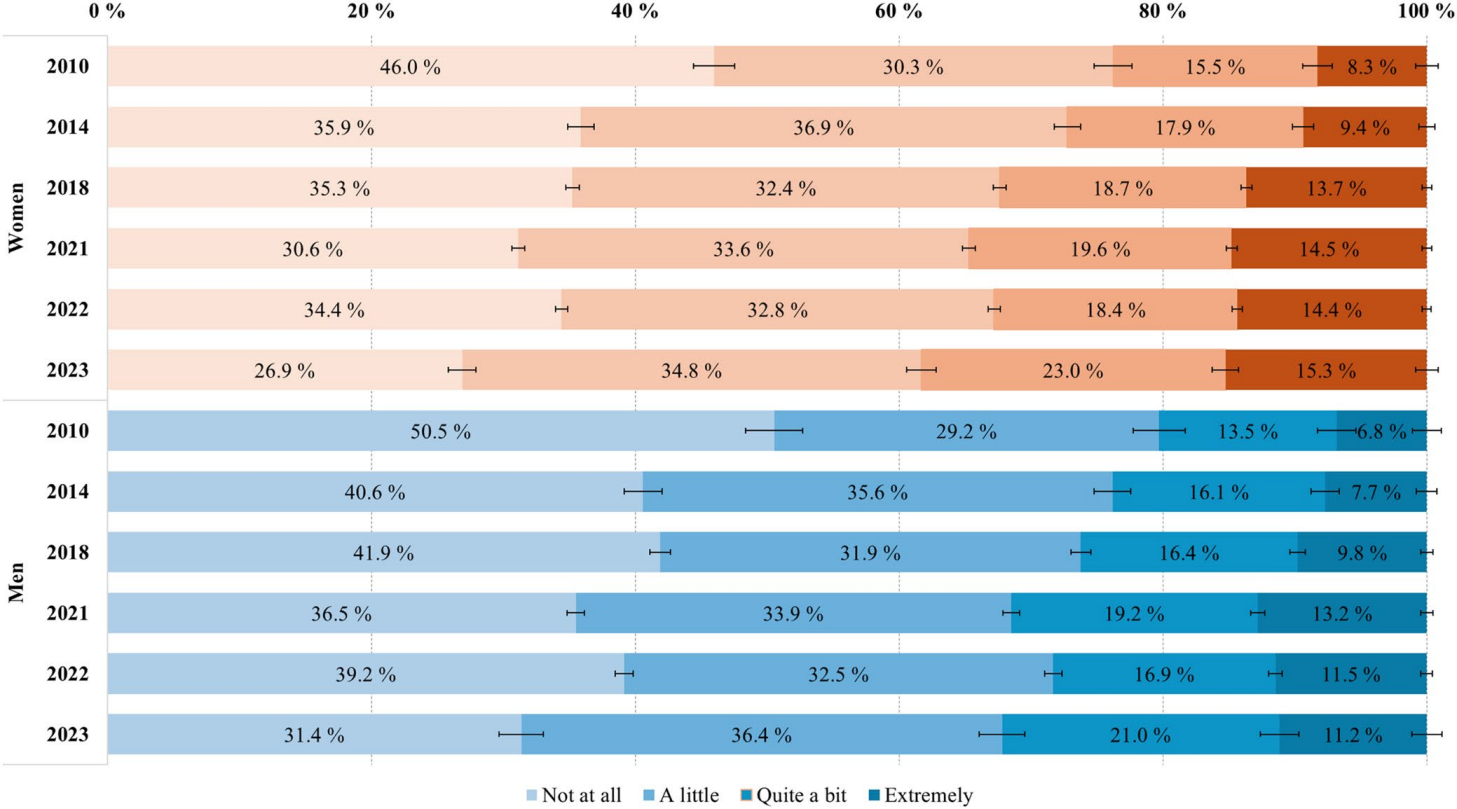
Distribution of responses to the difficulties initiating or maintaining sleep (DIMS) item from the Hopkins Symptom Checklist (HSCL), stratified by sex, among university students from 2018 to 2023. Error bars represent 95% confidence intervals.

Figure 1 illustrates the prevalence of DIMS, defined as responding ‘quite a bit’ or ‘extremely’ on the HSCL sleep item, among male and female students from 2010 to 2023. DIMS prevalence steadily increased in both sexes, with consistently higher rates among women. Among females, prevalence rose from 23.8% in 2010 to 32.4% (2018), 34.1% (2021), 32.8% (2022) and peaked at 38.3% in 2023. Among males, rates increased from 20.3% (2010) to 26.2% (2018), 32.4% (2021), 28.4% (2022) and 32.2% (2023), respectively.

Notably, increases occurred in both the ‘quite a bit’ and ‘extremely’ response categories. Among women, the proportion endorsing ‘extremely’ rose from 8.3% (2010) to 15.3% (2023), while ‘quite a bit’ increased from 15.5% to 23.0% over the same period. Among men, ‘extremely’ rose from 6.8% to 11.2%, and ‘quite a bit’ from 13.5% to 21.0%. These parallel trends suggest that not only has the prevalence of DIMS increased over time, but both the frequency and perceived severity of sleep problems have risen in tandem.

### 3.5 Logistic Regressions

Compared to the baseline year 2010, the ORs for reporting significant DIMS were significantly elevated in all subsequent waves for both sexes. Among females, ORs ranged from 1.20 (95% CI: 1.10–1.31) in 2014 to 1.99 (1.82–2.17) in 2023. Among males, corresponding ORs ranged from 1.23 (1.08–1.40) to 1.87 (1.63–2.13).

Focusing on the period from 2018 to 2023, which includes the years before (2018), during (2021) and after (2022–2023) the COVID‐19 pandemic, a consistent increase was observed. Among females, the OR was significantly higher in 2021 compared to 2018 (OR = 1.10, 95% CI: 1.03–1.18), remained unchanged between 2018 and 2022 (1.02, 0.99–1.10) and increased further from 2022 to 2023 (1.27, 1.17–1.37). The OR in 2023 was significantly higher than in both 2018 (1.40, 1.30–1.51) and 2021 (1.27, 1.18–1.37). Among males, the OR increased from 2018 to 2021 (1.29, 1.15–1.44), decreased from 2021 to 2022 (0.86, 0.75–0.97) and increased again from 2022 to 2023 (1.20, 1.05–1.38). The OR in 2023 was significantly higher than in 2018 (1.32, 1.17–1.48), while no significant difference was observed between 2021 and 2023 (1.03, 0.97–1.09).

## 4 Discussion

This study used repeated cross‐sectional data from the SHoT surveys (2010–2023) to examine long‐term trends in sleep health among Norwegian university students. Across the 2018, 2021 and 2022 waves, we observed marked changes in key sleep parameters, including longer SOL, more WASO and reduced sleep efficiency during the pandemic, with only partial recovery thereafter. At the same time, the prevalence of both DIMS and DSM‐5‐based insomnia disorder symptoms increased substantially across the six survey waves, with the steepest rises during the pandemic years. Importantly, these changes occurred alongside relatively stable average sleep duration, indicating that worsening sleep health was driven primarily by impairments in sleep continuity and efficiency rather than reductions in total sleep time. Overall, this pattern seems to reflect findings from the general population attesting to no clear trend regarding sleep duration (Bin et al. 2012), however, a trend toward an increase in sleep problems seems to be present (Pallesen et al. 2014; Wang et al. 2023; Holstein et al. 2020). By 2023, sleep problems had not returned to prepandemic levels, suggesting that these disruptions reflect not just a temporary deviation but a sustained shift in student sleep health.

These findings are consistent with recent studies demonstrating a worsening of student sleep health during the COVID‐19 pandemic. For example, a longitudinal study in Canada found that probable insomnia among university students increased from 18.1% to 29.7% during the pandemic (King et al. 2023). Similarly, a global survey across 59 countries reported that more than half of respondents delayed their bed and wake times, with over a third experiencing greater sleep disturbances at the start of the pandemic (Yuksel et al. 2021). A meta‐analysis of 18 studies confirmed a modest decline in sleep quality among high school and university students during the pandemic (Correa et al. 2024). Similarly, a systematic review attested to an increase in sleep problems among undergraduate students during the pandemic compared to prepandemic levels (Valenzuela et al. 2023). Ferreira‐Souza et al. (Ferreira‐Souza et al. 2023) further highlighted how COVID‐19‐related disruptions led to increased sleep variability and prolonged sleep latency, patterns that closely mirror our findings. Building on these studies, a key contribution of the present study lies in its long‐term perspective. While previous studies have primarily assessed short‐term changes during the pandemic, our results, covering both prepandemic, pandemic and postpandemic states, show that sleep problems among students were already increasing in the years preceding COVID‐19, and that these problems not only persisted but continued to worsen in the postpandemic period.

The underlying causes of these worsening sleep trends are likely multifactorial and should be interpreted within a broader psychosocial and structural context. Based on a review of students' health during the pandemic, Zarowski et al. argue (Zarowski et al. 2024) that students with a history of mental health issues and other comorbidities prior to the pandemic had worse sleep outcomes compared to healthy individuals. The increase in sleep problems over time may also partly be related to an ongoing trend. University students today face increasing academic, social and financial demands, which may contribute to elevated stress levels and irregular daily routines. The parallel increase in mental health problems and sleep problems is notable, and mental health problems and sleep are closely interconnected, potentially acting as mutually reinforcing processes. Several structural factors may also play a role. For instance, the expansion of higher education in Norway, reaching 37.9% of adults in 2024, up from 34.6% in 2019 (Statistics Norway 2025), has increased the diversity of the student population. While this expansion offers broader educational opportunities, it may also include more individuals from underrepresented backgrounds who may be at greater risk of sleep problems (Jackson 2018). These trends could also be interpreted in light of broader macroeconomic shifts. In Norway, county‐level data indicate a roughly 10‐percentage‐point increase from 2019/20 to 2023 in the share of young adults reporting difficulty making ends meet (Knapstad, Nilsen, et al. 2024; Knapstad, Nes, et al. 2024). Given the pronounced social gradient in sleep difficulties (Papadopoulos and Etindele Sosso 2023), declines in financial security may contribute to higher prevalence, exacerbate stress and widen inequalities in sleep health.

The shift toward digital learning during the pandemic may have further increased screen exposure and circadian disruption (Cabral et al. 2022). In addition, the temporary loss of structured schedules and diminished opportunities for physical activity likely contributed to the deterioration in sleep timing and quality during the pandemic (Sivertsen, Vedaa, et al. 2019; Yuksel et al. 2021; Sivertsen et al. 2025). This interpretation is supported by the SHoT2021 data, which showed delayed bedtimes and risetimes, as well as increases in SOL and WASO. Despite these changes, mean sleep duration remained largely unchanged, underscoring that sleep problems seem to be characterised by poorer sleep continuity, rather than reduced sleep opportunity. In addition, students reported pronounced levels of social jetlag, with nearly 2 h' discrepancy between weekday and weekend sleep timing. This degree of circadian misalignment is clinically relevant, as social jetlag has been linked to impaired mood, cognition and increased cardiometabolic risk (Wong et al. 2015; Islam et al. 2020). Although some sleep parameters partially reverted by 2022, key indicators such as sleep efficiency and SOL did not return to prepandemic levels, suggesting persistent dysregulation of sleep patterns. Furthermore, it is unlikely that changes in reporting behaviour alone explain these findings. Sleep complaints generally carry relatively low stigma compared to mental health symptoms, and discussions around sleep and well‐being have become more normalised in recent years (Cybulski et al. 2021; Stupinski et al. 2022; Lipson et al. 2019). While declining stigma and increased awareness may have contributed to greater willingness to report, this effect would most likely manifest as increases in milder symptom categories that are particularly sensitive to changing social norms. In contrast, we observed the largest increases in the most severe response category (‘extremely’), suggesting a genuine rise in symptom severity rather than a reporting/secular artefact. This interpretation is supported by concurrent increases in behavioural indicators of sleep problems, including prolonged sleep latency and reduced sleep efficiency, as observed in SHoT data. Taken together, these findings suggest that broader psychosocial or structural factors, rather than shifts in willingness to report, are likely contributing to the observed deterioration in student sleep health.

The present findings carry important implications for both public health and higher education. Sleep problems among university students have been consistently linked to a range of negative outcomes, including impaired academic performance, reduced psychological well‐being and elevated risk for long‐term physical health problems, mental health problems and safety hazards (Owens et al. 2017). The observed increases in both prevalence and severity of sleep problems, particularly following the COVID‐19 pandemic, underscore the importance of considering student sleep health within broader institutional and public health strategies. From a public health perspective, there is growing recognition that traditional sleep hygiene education, while useful, may be insufficient as a standalone approach (Ruan et al. 2025). More comprehensive and multilevel strategies may be warranted and appear more promising than simpler approaches (Tadros et al. 2025). Intervention at a more societal level could include structural adjustments, such as later class start times to better accommodate circadian preferences in young adults, as well as population‐level initiatives aimed at promoting healthy sleep behaviours and reducing environmental barriers to sleep. However, the effectiveness and feasibility of such measures in higher education settings require further investigation.

Finally, while this study benefits from repeated large‐scale survey data collected over a 13‐year period, several limitations need to be acknowledged. First, all sleep variables were based on self‐report, which may be subject to recall bias or reporting inaccuracies. Second, response rates across the survey waves were moderate, and differences by sex, age or other characteristics may have introduced some selection bias. Women were consistently overrepresented, which may limit generalisability, and students experiencing sleep or health problems may have been more likely to participate, potentially inflating prevalence estimates. However, because most analyses were stratified by sex, the observed temporal trends within women and men are less likely to be affected by sex imbalance in participation. The demographic composition of the SHoT samples was broadly comparable to the national student population and remained relatively stable across waves. Importantly, the primary aim of this study was population‐level surveillance of temporal trends rather than precise estimation of absolute prevalence, and such trend analyses are generally less sensitive to selection bias provided that recruitment procedures and participation patterns are reasonably consistent over time (Keiding and Louis 2016). Although the core recruitment approach was largely stable across waves (primarily direct email and SMS invitations supported by student welfare organisations), it cannot be ruled out that changes in participation propensity within demographic strata over time may have influenced the observed trends. Third, although a subset of participants took part in both the 2022 and 2023 surveys, the study was overall not designed as a longitudinal cohort. The 2023 data collection involved selective re‐invitation and eligibility restrictions, and analyses treated all survey waves as independent cross‐sections. This approach prioritises population‐level surveillance over within‐person change and may underestimate individual trajectories in terms of sleep health. Moreover, the repeated cross‐sectional design limits mechanistic interpretation of the observed patterns. Future studies using longitudinal designs, objective sleep assessments (e.g., actigraphy or polysomnography), and diagnostic interviews would be valuable for validating and extending these findings.

In conclusion, sleep problems among university students have increased steadily since 2010, with a marked rise during the COVID‐19 pandemic and further worsening by 2023. These trends reflect not only increasing prevalences but also a deterioration in sleep quality and continuity, despite stable sleep duration, particularly among female students. The findings underscore the importance of addressing student sleep as a core component of health promotion and academic support, and point to the need for sustained surveillance and targeted interventions in the years ahead.

## Author Contributions

B.S. is the guarantor of the study. B.S. contributed to the initiation, planning and design of the SHoT data collections. B.S. conducted the statistical analyses on the survey data, carried out the literature review and led the writing of the manuscript. All authors, A.G.H., S.P., Ø.V. and M.H., contributed with input on the design and analytical plan, interpretation of results, writing of the first draft and critical revision of the manuscript and analyses. All authors approved the final manuscript for submission.

## Funding

SHoT2018 and SHOT2022 have received funding from the Norwegian Ministry of Education and Research (2017) and the Norwegian Ministry of Health and Care Services (2016).

## Conflicts of Interest

The authors declare no conflicts of interest.

## Data Availability

Norwegian data protection regulations and GDPR impose restrictions on sharing individual participant data. However, researchers may request access to survey participant data by contacting the publication committee (borge.sivertsen@fhi.no). Approval from the Norwegian Regional Committee for Medical and Health Research Ethics (https://helseforskning.etikkom.no) is required for access to the data. The dataset is administered by the NIPH, and guidelines for data access can be found at https://www.fhi.no/en/more/access‐to‐data.
